# The effect of prebiotics on the gut microbiota in patients treated with atypical antipsychotics: a pilot study

**DOI:** 10.1192/j.eurpsy.2026.10529

**Published:** 2026-07-31

**Authors:** E. J. Giltay, N. J. de Bles, N. Rius-Ottenheim, J. P. Bogers

**Affiliations:** 1 Psychiatry, Leiden University Medical Center (LUMC), Leiden; 2 Intensive Care Clinics, Mental Health Organization (GGZ) Rivierduinen, Oegstgeest, Netherlands

## Abstract

**Introduction:**

Atypical antipsychotic (AAP) drugs are prescribed to patients with psychotic and other mental disorders. A common adverse effect is metabolic syndrome (MetS), often via weight gain and dyslipidemia. Besides lifestyle factors, drug-induced gut dysbiosis has been proposed as a mechanism. Dysbiosis is often marked by reduced *Bifidobacterium*, yet its causal role in MetS remains unclear.

**Objectives:**

Our aim was to evaluate the relative change in the proportion of bifidobacteria within the total fecal bacterial community, with the hypothesis that supplementation would lead to an increase.

**Methods:**

We included psychiatric patients with BMI > 25 kg/m², using long-term atypical antipsychotics in a pilot study. Following a 4-week run-in period, participants received a daily dose of the prebiotic combination of galacto-oligosaccharides (GOS) and 2’-fucosyllactose (2’-FL) (7.0 g Biotis® GOS + 0.7 g 2’-FL) for 6 weeks, with a washout of 4 weeks thereafter. The primary outcome was change in fecal bifidobacteria. Secondary outcomes included mental health (e.g., EQ-5D utility score) and metabolic syndrome measures. Fecal microbiome composition was assessed by 16S rRNA gene sequencing of the V4 region on the Illumina NovaSeq6000 platform.

**Results:**

Twenty-one participants using AAP (clozapine; n=9) were included (mean age 43.4 years; 67% male); of whom 76% had a psychotic disorder. Gut microbiome composition shifted from baseline to week 4, with stability thereafter. *Faecalibacterium* increased significantly (p=0.03) during supplementation, while diversity indices remained unchanged. *Bifidobacterium* increased non-significantly (p=0.10) during supplementation, but the overall increase from baseline to week 10 was statistically significant (change = 0.098; SE = 0.030; p = 0.002). This may be explained by the possibility that some participants may have unintentionally started their prebiotics already during the run-in phase. No serious adverse events occurred. Metabolic assessments (BMI, WHR, RR) did not change during supplementation. Subjective scales showed slight favorable changes, but none reached statistical significance, except for the EQ-5D utility score (QoL), which improved significantly (p = 0.01).

**Image 1:**
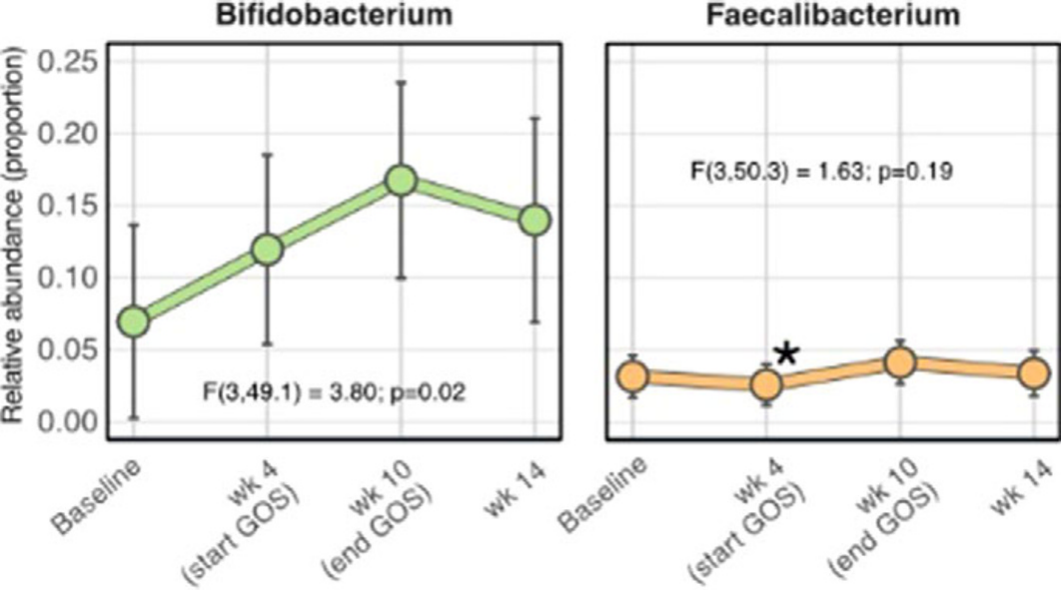
Image 1: Long description.

**Conclusions:**

Prebiotic supplementation was well tolerated and associated with some favorable shifts in gut microbiota, supporting its potential as an adjunctive strategy to mitigate antipsychotic-associated dysbiosis.

**Disclosure of Interest:**

E. Giltay Grant / Research support from: The Study was funded by a grant from FrieslandCampina, N. de Bles: None Declared, N. Rius-Ottenheim: None Declared, J. Bogers: None Declared.

## Long descriptions

### Image 1: Long description

A two-panel line graph. The y-axis for both panels is Relative abundance (proportion) ranging from 0.00 to 0.25. The x-axis for both panels shows four time points: Baseline, wk 4 (start G O S), wk 10 (end G O S), and wk 14.

Left Panel: Titled Bifidobacterium. A green line with circular markers shows a steady increase from a baseline of approximately 0.07 to a peak at wk 10 of approximately 0.17, followed by a slight decrease to 0.14 at wk 14. Large vertical error bars are present at each point. Statistical text at the bottom right reads F(3,49.1) = 3.80; p=0.02.

Right Panel: Titled Faecalibacterium. An orange line with circular markers remains relatively flat, fluctuating between 0.025 and 0.04 across all time points. Small vertical error bars are present. An asterisk is positioned above the wk 4 data point. Statistical text in the upper center reads F(3,50.3) = 1.63; p=0.19.

Navigate back to Image 1:.

